# Expression of MUC-2, MUC-6, NAPE-PLD, IL-6 and IL-13 in Healthy and Metaplastic Bronchial Epithelium

**DOI:** 10.3390/diseases11010005

**Published:** 2022-12-27

**Authors:** Elizabeta Lohova, Mara Pilmane

**Affiliations:** Institute of Anatomy and Anthropology, Riga Stradins University, Kronvalda Boulevard 9, LV-1010 Riga, Latvia

**Keywords:** metaplasia, bronchial epithelium, dysplasia, stratified squamous epithelium, lungs

## Abstract

*Background:* The normal tissue structure of the respiratory system is necessary to provide adequate protection of the airways and lungs. Prolonged exposure to trigger factors can result in adaptive mechanism activation and lead to the development of chronic pulmonary diseases or even dysplastic changes. *Materials and methods*: Respiratory system material with a pseudostratified ciliated epithelium was obtained from 12 patients (aged 16 to 95), and material with a stratified squamosa epithelium was obtained from six patients (aged 23 to 93). Routine staining was performed, and an immunohistochemistry was conducted for MUC-2, MUC-6, NAPE-PLD, IL-6 and IL-13. *Results:* Inflammatory processes were not detected in any of the specimens. A number of correlations were identified, with the most important being a strong positive correlation for IL-13 between the alveolar epithelium and alveolar macrophages and a strong positive correlation for IL-6 between the alveolar epithelium and alveolar macrophages in the stratified squamous epithelium group. We also detected a statistically significant difference in IL-6 in alveolar macrophages. *Conclusions:* There were no signs of dysplastic changes in either group. Increased secretion of IL-13 in the stratified squamous epithelium group shows its involvement in metaplastic changes in the bronchial epithelium. The secretion of atypical factors by hyaline cartilage demonstrates its plasticity and adaptability.

## 1. Introduction

The normal tissue structure of the respiratory system is required to provide adequate protection of the airways and lungs and ensure that all the pathogens are destroyed by the immune system or removed from the respiratory tract, and normal respiratory function occurs. The respiratory system is exposed to various trigger factors throughout life. Unfortunately, prolonged exposure to triggers can lead to various factors’ expression changes and alter the normal structure of tissues, leading to malfunctioning of the respiratory system’s defense mechanisms. Cellular adaptation such as hyperplasia, atrophy, hypertrophy and metaplasia of tissue can lead to chronic disease development: asthma and chronic obstructive pulmonary disease (COPD) [1]. It is possible to observe dysplastic changes in respiratory system tissue; these can lead to the development of tumors. Specific tissue factors have been reported to be connected with such changes.

Mucins (MUC) represent a heterogenous family of large complex glycoproteins and comprise an apomucin (polypeptide core) and carbohydrate chains linked to threonine and serine by O-glycosidic bonds [2,3]. They are synthesized and secreted by specialized cells of the epithelium, including goblet cells and cells of mucous glands, to protect epithelial tissues from exposure to different factors not only in the respiratory system but also in other organ systems of the organism, including the stomach, intestine, gall bladder, seminal vesicles, pancreatic ductules and periductal glands of the common bile duct [4,5]. MUC-5AC and MUC-5B are the main types of mucins secreted in normal respiratory epithelium; increased secretion of MUC-2 and MUC-6 is mainly observed during inflammation, and functional and morphological changes of the epithelium [6,7,8].

MUC-2 is a heavily glycosylated protein that is encoded by the MUC-2 gene located on 11p15.5 [3]. MUC-2 is a major part of intestinal mucins and most often found in the small intestine and colon; decreased expression of MUC-2 has been reported in non-mucinous colonic cancer [3]. It is also one of the four secreted mucins of the lungs; however, the amount of MUC-2 is minimal compared to MUC-5AC and MUC-5B [6,7]. Although the expression of MUC-2 in healthy lungs is decreased, relevant studies have demonstrated increased MUC-2 expression during acute and chronic inflammatory processes in the lungs. Li et al. in 1997 and Dohrman et al. in 1998 observed that MUC-2 secretion can be increased by a variety of Gram-positive (*Staphylococcus aureus, Staphylococcus epidermidis, Streptococcus pyogenes*) and Gram-negative (*Pseudomonas aeruginosa, Escherichia coli*) bacteria in the surface epithelium, as well as in the submucosal glands of human bronchial explants [8,9]. Bacteria induced MUC-2 synthesis and secretion is mainly associated with the lipopolysaccharide (LPS) connection with the epithelium; however, lipoteichoic acid and flagellin are known to stimulate MUC2 expression [6,8]. Not only can bacteria induce MUC-2 expression, but also cytokines like interleukin 1β (IL-1β), IL-6, IL-13 and TNF-α [6,10,11]. Increased secretion of MUC-2 was also observed in the metaplastic epithelium of airways, in chronic pulmonary diseases (asthma, COPD, cystic fibrosis) and lung cancers [10,12,13]. 

MUC-6 is a high molecular weight gel-forming mucin encoded by the MUC-6 gene, which is located on chromosome 11p15.5, only 38.5 kb apart from the MUC-2 gene [14]. Typically, MUC-6 secretion at high levels can only be found in healthy tissues of the stomach and gall bladder [5,15]. Indeed, increased expression of MUC-6 was detected in pulmonary, intestinal, colonic and mammary adenocarcinoma; it is not expressed in corresponding normal tissue [16,17,18]. Moreover, the levels of MUC-6 expression significantly increased in the progression from atypical adenomatous hyperplasia through to bronchioloalveolar carcinoma and adenocarcinoma with mixed subtypes [19]. Relevant studies have demonstrated increased expression of gastric MUC-6 after stimulation with IL-13 and IL-6 but decreased MUC-6 expression in patients with *Helicobacter pylori* infection [10,11,20,21].

As mentioned above, high-level expression of MUC-2 and MUC-6 is found in normal intestinal tissue; the elevated secretion of these two mucins in pulmonary and mammary tissue is most likely related to tissue metaplasia or even dysplasia.

Although the respiratory system’s defense mechanisms are complex, the expended influence of trigger factors leads to cellular adaptation, dysplasia and changes in protein expression, which are associated with the secretion of different cytokines. Two of these cytokines are IL-6 and IL-13, which are involved in the stimulation of MUC-2 and MUC-6 synthesis and secretion.

IL-13 is a pleiotropic cytokine secreted by different types of immune cells, mainly by Th2 cells [22]. The expression of IL-13 is stimulated by different triggers (allergens, viruses, IL-6 and others) and associated with inflammation and respiratory system tissue remodeling, such as goblet cell hyperplasia, mucus hypersecretion, airway hyperresponsiveness and fibrosis [23]. Donlan et al. in 2021 demonstrated IL-13 involvement in the development of a more severe form of Covid-19 [24]. Moreover, IL-13 plays a crucial role in mucous cell metaplasia and enhanced mucin production. IL-13 induces production of tumor growth factor α (TGFα) in epithelial cells, which results in mucous cell metaplastic changes [11,25]. Direct stimulation of airway epithelial cells by IL-13 induces selective MUC-5AC secretion, and stimulation of calcium-activated chloride channel 1 (hCLC1/Gob5) leads to the expression of gel-forming mucins like MUC-2, MUC-6, MUC-5AC and MUC-5B [11,25].

IL-6 is a proinflammatory mediator with functional pleiotropy and plays an important role in host defense [26]. It is produced by fibroblasts, mesenchymal cells and endothelial cells, but the main production of IL-6 is associated with monocytes and macrophages [27,28]. The functions of IL-6 involve increasing of fibroblast apoptosis, macrophage phagocytic activity and epithelial survival by extracellular matrix stabilization [27]. Moreover, IL-6 stimulates mast cells and induces the release of T-helper type-2 (Th2) cytokines such as IL-4, IL-5, IL-10 and IL-13 [26]. Once the trigger factor is removed, it is necessary to stop IL-6 production to provide balance in the lung environment. Relevant studies have shown that prolonged production of IL-6 can lead to autoimmune and inflammatory disease development, including rheumatoid arthritis, multiple myeloma, renal carcinoma and pulmonary adenocarcinoma [1,29,30]. An increased effect of IL-6 on MUC-2, MUC-5 and MUC-6 expression was reported in pulmonary and colon malignancies [10,29,30]. Prolonged secretion of IL-6 also indirectly stimulates expression of mucins by inducing the release of IL-10 and IL-13 [30]. These cytokines are involved in the development of metaplasia and increased secretion of mucins [11,30].

NAPE-PLD (N-acyl-phosphatidylethanolamine-hydrolyzing phospholipase D) is a phospholipase D-type enzyme that is encoded by the NAPE-PLD gene located on chromosome 7q22.1 [31,32]. The biological purpose of the NAPE-PLD enzyme relates to the functions of N-acylethanolamines, also known as fatty acid amides (FAAs). The types of NAEs and their roles are as follows: anandamide: activates cannabinoid receptors; N-palmitoyl-ethanolamine: anti-inflammatory action; and N-oleoyl-ethanolamine: regulates appetite, fat metabolism and cancer cell proliferation [33,34,35]. Wenzel et al. in 2013 demonstrated enhanced expression of the NAPE-PLD protein in hypoxia [36]. Studies on NAPE-PLD synthesis and secretion after LPS stimulation are controversial. Liu et al. in 2003 demonstrated increased expression of NAPE-PLD after stimulation by LPS; meanwhile, Zhu et al. in 2011 showed a suppressive effect of LPS on NAPE-PLD secretion and activity [37,38]. Relevant studies have shown a connection between the expression of NAPE-PLD and IL-6. Reduced expression of IL-6 was observed after inhibition of NAPE-PLD, which suggests NAPE-PLD involvement in the regulation of IL-6 expression [39]. The highest expression of NAPE-PLD was found in the gastrointestinal tract and brain [36,39]. Expression levels are considered medium in lung tissue; NAPE-PLD expression was found in the bronchial epithelium and alveolar macrophages [36,39]. While the expression of NAPE-PLD is normally found in healthy tissues, increased protein expression was also found in dysplastic tissues, such as lung, endometrial, breast, ovarian, renal, pancreatic, prostate, brain and colon cancers [39,40,41]. Moreover, the most intense expression of NAPE-PLD was observed in the basal components of glandular cells [40]. These findings demonstrate the possible use of NAPE-PLD as a marker to detect dysplastic changes in tissues, especially glandular tissue. 

Mucins MUC-2 and MUC-6, NAPE-PLD and interleukins are not found in healthy lung tissue. IL-6 and IL-13 are associated with chronic pulmonary diseases; prolonged expression can lead to metaplastic or even dysplastic changes [10,11,25,29]. Meanwhile, expression of mucins and NAPE-PLD in lung tissue are markers for dysplastic changes [9,11,13,14,39,40,41].

On the basis of the above, our working hypothesis was that there is a difference in the distribution and appearance of mucins, cytokines and enzymes in lungs with a stratified squamosa epithelium and pseudostratified ciliated epithelium. Thus, the aim of this study was to compare the appearance and relative number of mucins, cytokines and enzyme expression in lungs with a healthy unchanged (pseudostratified ciliated) and changed (stratified squamous) bronchial epithelium.

## 2. Materials and Methods

### 2.1. Material Characteristics of Subjects

This study was approved by the Ethics Committee for Clinical Research of Medicine and Pharmaceutical Products at Pauls Stradins Clinical University Hospital Development Foundation in Latvia (Nr.230113-17L, 2013). The study was conducted at the Institute of Anatomy and Anthropology, Latvia. The respiratory system material was obtained from 18 patients. Twelve samples were obtained from 9 males in the age range 16 to 94 and 3 females in the age range 55 to 95, and contained pseudostratified ciliated epithelium. Six specimens were obtained from males in the age range 23 to 93 containing stratified squamous epithelium. Tissues were not associated with inflammation or any other pathology. The material was collected during the postmortem autopsy from persons who died in accidents and/or due to reasons not connected to respiratory tract disease or lung surgery. The lung tissue material used in the study was obtained at autopsy 12–24 h after the biological death of patients.

For patient selection, to exclude as many co-factors and confounders as possible, inclusion and exclusion criteria were developed.

The inclusion criteria were as follows: (1) patient older than seven years (age when the lungs are considered morphologically mature and corresponding to the lung morphology of an adult individual); (2) the obtained lung tissue sample histologically complies with the requirements of the tissue sample as determined in the study and contains bronchial material (and/or lung parenchymal material).

The exclusion criteria were as follows: (1) pathological finding in the lung tissue material (inflammatory cell infiltration, chronic inflammation, etc.); (2) acute or chronic lung disease in medical history; (3) lung oncological disease; (4) no bronchial and/or pulmonary material found in the tissue section; (5) living and/or working environment that affects the lungs; (6) smoking habits during life. The causes of death were accidents or fatal self-harm (trauma to body parts and organs, suicides that were not compatible with life). The causes of death of three individuals were associated with acute cardiovascular failure and/or ischemic heart disease (cardiac arrest).

### 2.2. Immunohistochemical Analysis

The tissue specimens were fixed in a mixture of 2% formaldehyde and 0.2% picric acid in 0.1 M phosphate buffer (pH 7.2). Afterwards, they were rinsed in Tyrode buffer containing 10% saccharose for 12 h, embedded into paraffin and cut into 3–4 μm thin sections. The sections were stained with hematoxylin and eosin for routine morphological evaluation. The Biotin-Streptavidin biochemical method was used for immunohistochemistry (IMH) to detect: mucin 2 (MUC-2) (1112207D, working dilution 1:100, Cell-MARQUE, USA); mucin 6 (MUC-6) (1129302G, working dilution 1:200, Cell-MARQUE, USA); N-acyl-phosphatidylethanolamine-hydrolyzing phospholipase D (NAPE-PLD) (sc-514372, working dilution 1:100, 1:300, 1:500, Santa Cruz Biotechnology Inc., Santa Cruz, CA, USA); interleukin 6 (IL-6) (sc-28343, working dilution 1:100, Santa Cruz Biotechnology Inc., Santa Cruz, CA, USA); interleukin 13 (IL-13) (sc-390576, working dilution 1:100, Santa Cruz Biotechnology Inc., Santa Cruz, CA, USA).

The stained slides were analyzed by light microscopy using non-parametric evaluation, which is widely used for the semiquantitative evaluation of data in morphology, by two morphologists acting independently [42,43]. The results were evaluated by grading the appearance of the positively stained cells in the visual field [43]. The designation was as follows: 0—no positive structures in the visual field; 0/+—occasional positive structures in the visual field; +—a few positive structures; +/++—a few to a moderate number of positive structures in the visual field; ++—a moderate number of positive structures in the visual field; ++/+++—moderate to numerous positive structures in the visual field; +++—numerous positive structures with the visual field; +++/++++—numerous to abundant positive structures in the visual field; ++++—abundant positive structures in the visual field.

For visual illustration, a Leica DM500RB digital camera and Microsoft Photo editor (version 19051.16210.0) were used.

### 2.3. Statistical Analysis

We used the non-parametric Mann-Whitney U test to compare the immunoreactive positive cell count between the pseudostratified ciliated epithelium group and stratified squamous epithelium group. 

Friedman's two-way analysis was performed for multiple comparisons of all factor immunoreactive cell count mean ranks in different tissue locations. Bonferroni adjustments were run for post-hoc tests.

We used Spearman’s rank correlation coefficient (ρ), whereby ρ = 0–0.3 was assumed to be a very weak correlation, ρ = 0.3–0.5 a weak correlation, ρ = 0.5–0.7 a moderate correlation, ρ = 0.7–0.9 a strong correlation, and ρ = 0.9–1 a very strong correlation. 

The statistical data processing was performed with IBM SPSS (Statistical Package for the Social Sciences) version 26.0. The significance level for all tests was selected as a *p*-value < 0.05 (5%).

## 3. Results

### 3.1. Tissue Routine Examination

**Figure 1:**
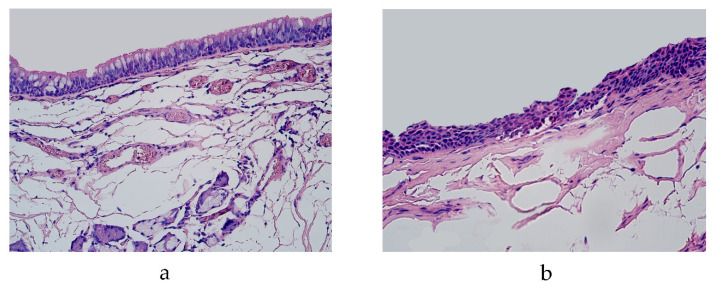
Micrograph of lungs routinely stained by hematoxylin and eosin. (**a**) Pseudostratified ciliated epithelium in the bronchi of 19-year-old male. ×200; (**b**) squamous stratified epithelium in the bronchi of 46-year-old male. ×250.

The lung tissue contained bronchial and lung parenchyma in all the specimens. We observed two different types of bronchial epithelium in the lung tissue specimens: pseudostratified ciliated epithelium and stratified squamous epithelium (Figure 1a,b). No histological changes associated with inflammatory processes were observed in any of the specimens.

### 3.2. Immunohistochemical (IMH) Data

We evaluated the expression of MUC-2, MUC-6, NAPE-PLD, IL-6 and IL-13 at six locations in the lung tissue: bronchial epithelium, connective tissue, cartilage, glands, alveolar epithelium and alveolar macrophages.

**Figure 2:**
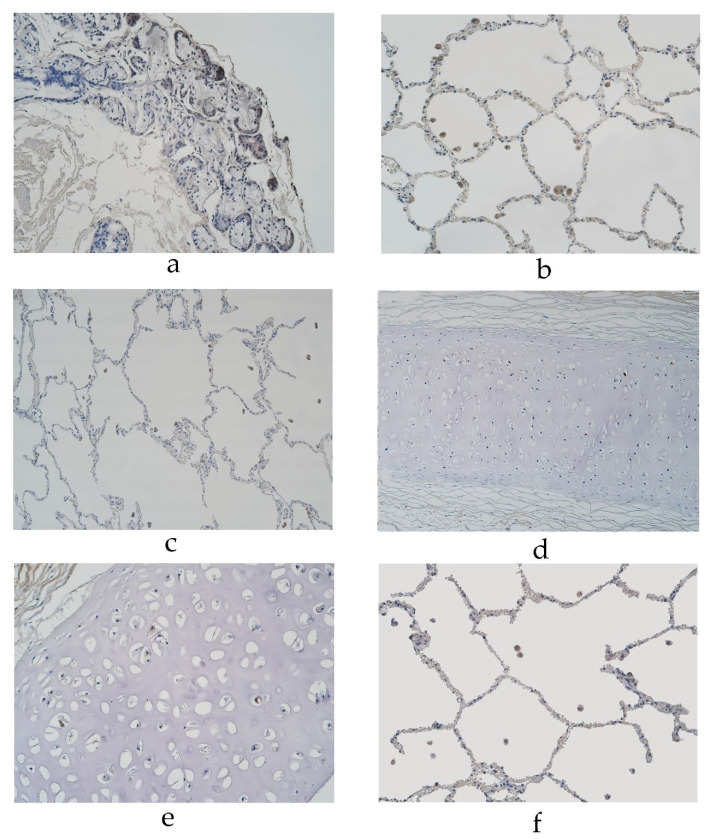
Immunohistochemical micrographs of the lungs. (**a**) MUC-2 positive cells in glands of 94-year-old male in the pseudostratified ciliated epithelium group. MUC-2 IMH, ×200. (**b**) A few MUC-2 positive alveolar macrophages in ac56-year-old male in the pseudostratified ciliated epithelium group. MUC-2 IMH, ×200; (**c**) A few to a moderate number of MUC-2 positive alveolar macrophages observed in a 23-year-old male in the stratified squamous epithelium group. MUC-2 IMH, ×200; (**d**) Occasional MUC-6 positive cells in cartilage of 55-year-old female in the pseudostratified ciliated epithelium group. MUC-6 IMH, ×200; (**e**) Occasional number of MUC-6 positive cells in cartilage of 67-year-old male in the stratified squamous epithelium group. MUC-6 IMH, ×200; (**f**) A few MUC-6 positive alveolar macrophages in 25-year-old male in the stratified squamous epithelium group. MUC-6 IMH, ×200.

In the pseudostratified ciliated epithelium group, the expression of MUC-2 was detected in the glands of a 29-year-old male in a few cells and a 94-year-old male in occasional cells (Table 1, Figure 2a). MUC-2 was also present in occasional alveolar macrophages in a 29-year-old male and in a few alveolar macrophages of 56- and 94-year-old males in the pseudostratified ciliated epithelium group (Table 1, Figure 2b). In the stratified squamous epithelium group, occasional positive cells were detected in the glands of a 46-year-old male and in a few alveolar macrophages of a 23-year-old male (Table 1, Figure 2c). We did not detect any MUC-2 positive cells in other locations of the lung tissue (bronchial epithelium, connective tissue, cartilage and alveolar epithelium). 

The cartilage demonstrated occasional MUC-6 positive cells in a 16-, 19- and 94-year-old male and 55-year-old female in the pseudostratified ciliated epithelium group (Table 1, Figure 2d). In the pseudostratified ciliated epithelium group, a few MUC-6 positive alveolar macrophages were detected in a 56- and 94-year-old male (Table 1). In the stratified squamous epithelium group, MUC-6 was present in occasional cartilage cells of a 67-year-old male and a few alveolar macrophages of a 23- and 25-year-old male (Table 1, Figure 2e,f). We did not detect any MUC-6 positive cells in other locations of the lung tissue (bronchial epithelium, connective tissue, glands or alveolar epithelium). 

**Figure 3:**
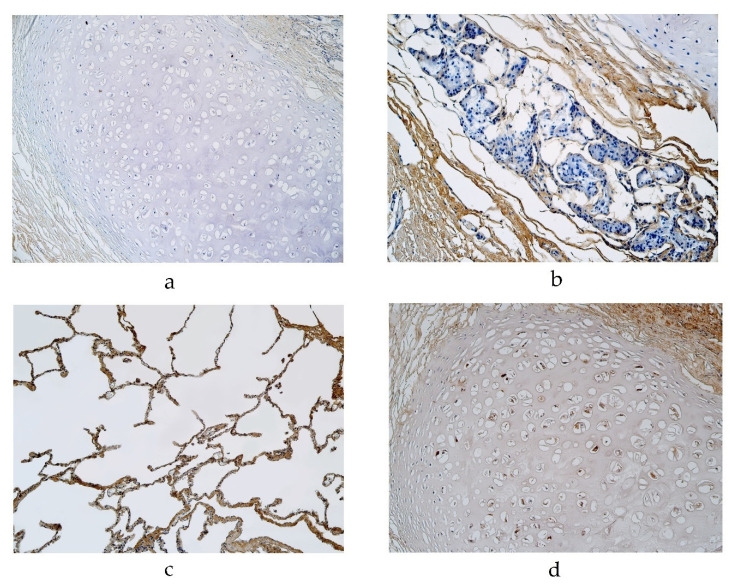
Immunohistochemical micrographs of the lungs. (**a**). Occasional NAPE-PLD positive cartilage in a 56-year-old male in the pseudostratified ciliated epithelium group. NAPE-PLD IMH, ×200; (**b**) Occasional NAPE-PLD positive structures in glands of a 94-year-old male in the pseudostratified ciliated epithelium group. NAPE-PLD IMH, ×200; (**c**) A few NAPE-PLD positive macrophages and a moderate number of positive alveolar epithelium of a 23-year-old male in the stratified squamous epithelium group. NAPE-PLD IMH, ×200; (**d**) A moderate number of positive structures in the cartilage of a 46-year-old male in the stratified squamous epithelium group. NAPE-PLD IMH, ×200.

The expression of NAPE-PLD was detected in a moderate number of cartilage and alveolar epithelium cells and a few to a moderate number of alveolar macrophages in a 29-year-old male in the pseudostratified ciliated epithelium group (Table 1). NAPE-PLD expression was detected in a 56-year-old male’s lung tissue with a few positive cells in cartilage, a few to moderate number of positive alveolar epithelium cells and a moderate number of positive alveolar macrophages (in the pseudostratified epithelium group) (Table 1, Figure 3a). In the pseudostratified ciliated epithelium group, a few positive structures of NAPE-PLD were detected in the glands of a 94-year-old male and a few to moderate number of positive structures were detected in the alveolar epithelium of a 95-year-old female (Table 1, Figure 3b). In the stratified squamous epithelium group, the expression of NAPE-PDL was detected in a 23-year-old male’s lung tissue, with a moderate number of positive structures in cartilage and alveolar epithelium cells and a few to a moderate number of positive alveolar macrophages (Table 1, Figure 3c). A moderate number of NAPE-PLD positive structures were detected in the cartilage of a 46-year-old male (Table 1, Figure 3d). We did not detect any NAPE-PLD positive cells in the bronchial epithelium or connective tissue. 

**Figure 4:**
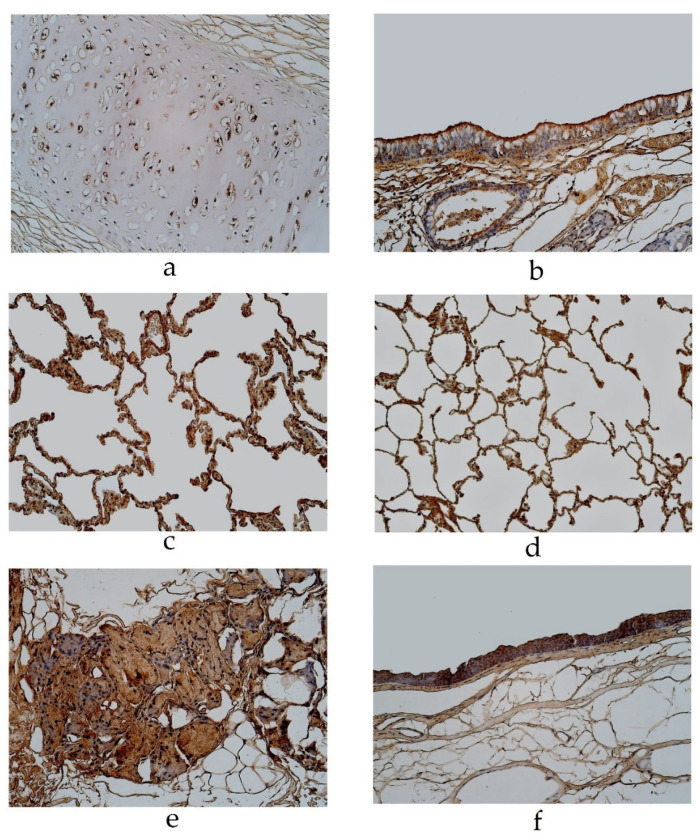
Immunohistochemical micrographs of the lungs. (**a**) Moderate to numerous number of IL-6 positive structures in the cartilage of a 94-year-old male in the pseudostratified ciliated epithelium group. IL-6 IMH, ×200; (**b**) A few IL-6 positive bronchial epithelial cells observed in a 19-year-old male of the group with the pseudostratified ciliated epithelium. IL-6 IMH, ×200; (**c**) A moderate number of IL-6 positive cells in the alveolar epithelium of a 95-year-old female in the pseudostratified ciliated epithelium group. IL-6 IMH, ×200; (**d**) A moderate number of IL-6 positive alveolar epithelium cells observed in a 25-year-old male of the group with the stratified squamous epithelium group. IL-6 IMH, ×200; (**e**) Moderate number of positive IL-6 cells in the glands of a 23-year-old male in the stratified squamous epithelium group. IL-6 IMH, ×200; (**f**) Moderate number of IL-6 positive bronchial epithelial cells in a 46-year-old male of the stratified squamous epithelium group. IL-6 IMH, ×200.

The highest expression of IL-6 was detected in cartilage, where its expression fluctuated from no positive structures to moderate to numerous positive structures (in the pseudostratified ciliated epithelium group) (Table 2, Figure 4a). Occasional IL-6 positive structures were detected in the bronchial epithelium, glands, alveolar epithelium and macrophages in the pseudostratified ciliated epithelium group (Table 2, Figure 4b,c). Connective tissue demonstrated occasional positive structures in a 19- and 56-year-old male (Table 2, Figure 4b). In the stratified squamous epithelium group, a few to a moderate number of IL-6 positive structures was detected in the alveolar epithelium and macrophages, but IL-6 positive structures were occasionally detected in the bronchial epithelium, cartilage and glands (Table 2, Figure 4d–f). We did not detect any positive structures in connective tissue (Table 2). 

**Figure 5:**
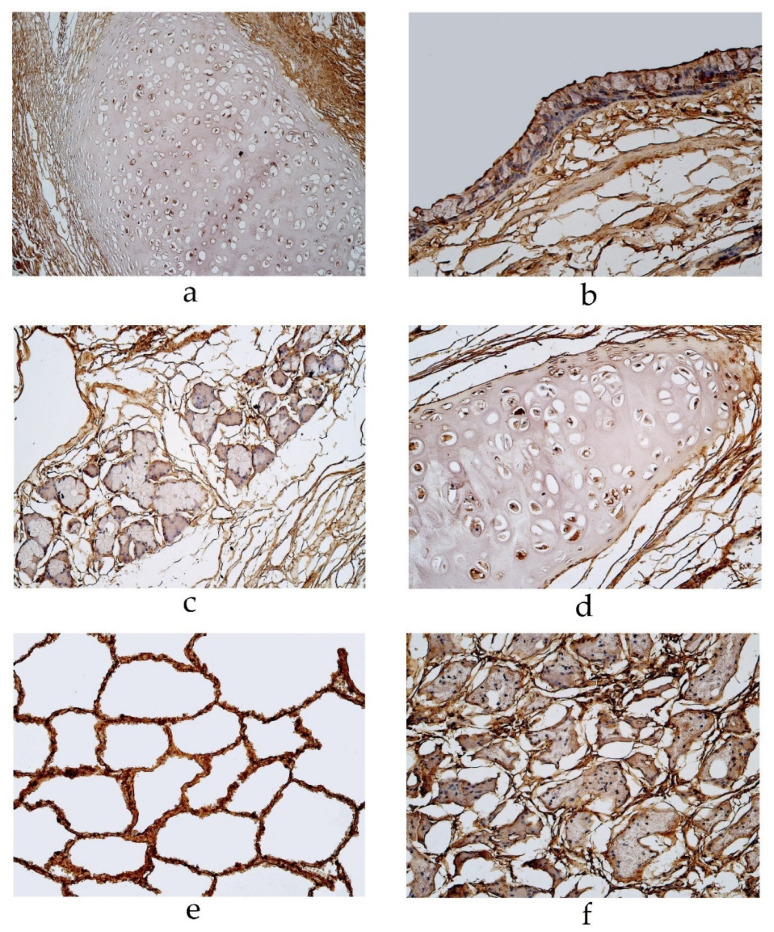
Immunohistochemical micrographs of the lungs (**a**) A moderate number of IL-13 positive cells observed in cartilage of a 19-year-old male in the pseudostratified ciliated epithelium group. IL-13 IMH, ×200; (**b**) A few IL-13 positive structures in the bronchial epithelium of a 29-year-old male in the pseudostratified ciliated epithelium group. IL-13 IMH, ×200; (**c**) A few IL-13-staind cells in the glands of a 95-year-old female in the pseudostratified ciliated epithelium group. IL-13 IMH, ×200; (**d**) A moderate IL-13-stained cells in the cartilage of a 23-year-old male in the stratified squamous epithelium group. IL-13 IMH, ×200; (**e**) A moderate number of IL-13-stained alveolar epithelium cells in an 86-year-old male of the stratified squamous epithelium group. IL-13 IMH, ×200; (**f**) A few to a moderate number of IL-13 positive weakly stained cells observed in the glands of an 86-year-old male in the stratified squamous epithelium group. IL-13 IMH, ×200.

In the pseudostratified ciliated epithelium group, IL-13 was observed in cartilage, where it showed a moderate number of positive structures (Table 2, Figure 5a). A few positive structures were detected in the bronchial epithelium, glands, alveolar epithelium and alveolar macrophages (Table 2, Figure 5b,c). Occasional IL-13 positive structures were observed in connective tissue (Figure 5b). In the stratified squamous epithelium group, a moderate number of IL-13 positive structures were observed in cartilage (Table 2, Figure 5d). IL-13 positive cells were observed in a few to a moderate number of the bronchial epithelia, alveolar epithelium and alveolar macrophages (Table 2, Figure 5e). IL-13 was present in occasional cells in connective tissue and glands (Table 2, Figure 5f). 

### 3.3. Statistical Analysis

A statistically significant difference between the pseudostratified ciliated epithelium group and stratified squamous epithelium group was noticed for IL-6 in the alveolar macrophages (*p* = 0.024) (Table 3).

In the pseudostratified ciliated epithelium group, a statistically significant overall difference was seen between all factors and in different tissue locations (*p* < 0.001). In particular, post-hoc analysis with Bonferroni adjustment revealed the mean rank comparison of the IL-13 immunoreactive cell count in cartilage was significantly higher than the MUC-2 immunoreactive cell count in the bronchial epithelium (*p* = 0.003), connective tissue (*p* = 0.003), cartilage (*p* = 0.003), alveolar epithelium(*p* = 0.003), and glands (*p* = 0.037); the MUC-6 immunoreactive cell count in the alveolar epithelium (*p* = 0.003), bronchial epithelium (*p* = 0.003), and connective tissue (*p* = 0.003); the NAPE-PLD immunoreactive cell count in the bronchial epithelium (*p* = 0.003), connective tissue (*p* = 0.003), and glands (*p* = 0.012); and the IL-6 immunoreactive cell count in connective tissue (*p* = 0.025).

The summary of the findings of the significance of the immunoreactive cell count of different factors in different tissue locations of the pseudostratified ciliated epithelium group is shown in Table 4.

In the stratified squamosa epithelium group, a statistically significant overall difference was seen between all factors and in different tissue locations (*p* < 0.001); however, post-hoc analysis with Bonferroni adjustment revealed no statistically significant difference between the immunoreactive cell count of different factors and different tissue locations.

Correlation analysis of the lung tissue group with the pseudostratified ciliated epithelium demonstrates a very strong positive correlation between IL-13 in alveolar epithelium and alveolar macrophages, as well as NAPE-PLD in cartilage and alveolar macrophages (Table 5).

A strong positive correlation was detected between IL-6 and IL-13 in the alveolar epithelium; IL-6 in the alveolar epithelium and IL-13 in the alveolar macrophages; IL-6 in the alveolar epithelium and IL-6 in the alveolar macrophages; and IL-6 and NAPE-PLD in the alveolar epithelium. Correlation analysis of the lung tissue group with the pseudostratified ciliated epithelium demonstrates a strong positive correlation between NAPE-PLD in the alveolar macrophages and NAPE-PLD in the alveolar epithelium; NAPE-PLD and MUC-2 in alveolar macrophages; NAPE-PLD and MUC-6 in alveolar macrophages; IL-6 and IL-13 in cartilage; NAPE-PLD in cartilage and NAPE-PLD in the alveolar epithelium; IL-13 and MUC-2 in alveolar macrophages; IL-13 and MUC-6 in alveolar macrophages; NAPE-PLD in cartilage and MUC-2 in alveolar macrophages; NAPE-PLD in cartilage and MUC-6 in alveolar macrophages; and IL-6 in alveolar macrophages and IL-13 in the alveolar epithelium (Table 5).

A moderate positive correlation was detected between MUC-2 in alveolar macrophages and IL-13 in the alveolar epithelium; MUC-6 in alveolar macrophages and IL-13 in alveolar epithelium; IL-6 in cartilage and IL-13 in connective tissue; IL-6 in bronchial epithelium and IL-6 in connective tissue; MUC-6 in cartilage and IL-6 in glands; IL-6 and IL-13 in alveolar macrophages; MUC-2 and NAPE-PLD in glands; and IL-6 and IL-13 in bronchial epithelium (Table 5).

Correlation analysis of the lung tissue group with the stratified squamous epithelium demonstrates a very strong positive correlation between IL-13 in the alveolar epithelium and IL-13 in alveolar macrophages and between IL-6 in the alveolar epithelium and IL-13 in connective tissue (Table 6).

A strong positive correlation was detected between MUC-6 in alveolar macrophages and IL-6 in the alveolar epithelium; IL-6 in the alveolar epithelium and IL-6 in alveolar macrophages; IL-6 in the bronchial epithelium and IL-6 in cartilage; and IL-13 in the bronchial epithelium and IL-13 in connective tissue (Table 6). 

## 4. Discussion

Metaplastic changes in respiratory system tissues are the result of complex processes associated with long-time trigger factor influences, chronic tissue irritation, changes in different protein (cytokines, mucins) expression and, in some cases, genetic predisposition [44]. 

Summarizing the results of our study, we observed expression of all factors in lung tissue of both study groups—in the pseudostratified epithelium and stratified squamous epithelium groups. However, alveolar macrophages were the main source of all factor synthesis in the stratified squamous epithelium group. 

Chronic inflammatory processes recruit immune cells, including macrophages, which leads to pro-inflammatory factor long-time synthesis and secretion with further formation of a microenvironment for metaplastic changes [44,45]. Relevant studies have shown macrophages involvement in tumor-growing processes such as the promotion of angiogenesis, involvement in extracellular matrix breakdown, remodeling of tissues and the promotion of tumor cell migration [45,46,47]. Notably, all the macrophages associated with supporting tissue changes in tumor development are similar to those in normal tissue development and wound healing [45]. Thus, tumor cells are using macrophages’ functions to promote tumor progression and metastases [45]. Two macrophage subtypes lead us to consider involvement in tumorigenesis: classical activated macrophages (M1) and alternatively activated macrophages (M2). The alternative activation of macrophages is induced by different cytokines, one of these is IL-13, which is produced by T lymphocytes, granulocytes and macrophages, and is associated with asthma development and lung tissue fibrosis [48]. This suggests IL-13 involvement in macrophage-induced changes in the respiratory system; however, M2 are normally involved in the decrease of inflammation, lung remodeling and production of anti-inflammatory factors, including IL-10, transforming growth factor β [49]. Another subtype of macrophages M1 is a potent effector cell that is influenced by different trigger factors, producing pro-inflammatory mediators like nitric oxide, tumor necrosis factor α, IL-6 and IL-12. [49,50]. Extended stimulation by IL-6 enhances expression of IL-13, MUC-2 and MUC-6, which play one of the main roles in metaplastic and dysplastic changes of tissue, including respiratory system tissue [10,23,29,30,50]. Normally, MUC-2 and MUC-6 expression is not found in healthy lung tissue and is associated with metaplastic and dysplastic changes [12,19]. As mentioned earlier, NAPE-PLD can be expressed by the bronchial epithelium and alveolar macrophages; however, increased levels of NAPE-PLD in the lungs also relates to metaplastic and dysplastic changes [36,39]. While the induction factors of NAPE-PLD secretion are not clearly known, cooperation between IL-6 and NAPE-PLD has been reported [39].

According to our data, the expression of MUC-2 and MUC-6 was not found in the bronchial epithelium of either group in any of specimens. As mentioned earlier, the expression of these factors is usually not found in healthy lung tissue [12,19]. Meanwhile, increased expression of mucins, especially MUC-2, in the bronchial epithelium is associated with adenocarcinoma and can be used as an oncological marker [12, 19]. Thus, we can conclude that the pseudostratified epithelium group and stratified squamous epithelium group did not develop dysplastic changes in the bronchial epithelium. We did not find the expression of mucins in the bronchial epithelium; we detected positive MUC-2 cells in the glands of some specimens in both study groups. As mentioned earlier, the expression of MUC-2 is not a normal finding in healthy lungs [19]. MUC-2 expression by glands in the respiratory system is detected in diseases like asthma, cystic fibrosis, COPD, chronic bronchitis, metaplasia and dysplasia [51,52]. The expression of these factors in glands can show some early changes in tissue which possibly will lead to pathology development or metaplastic or dysplastic changes. 

In our study, we detected some positive cells of NAPE-PLD in the glands and alveolar epithelium of both groups, but there were no immunoreactive cells in the bronchial epithelium. The NAPE-PLD expression level is generally very low or even undetectable in respiratory system tissue [36,39]. Relevant studies have observed increased NAPE-PLD expression during hypoxia [36]. Moreover, increased expression of NAPE-PLD in lung tissue can be associated with dysplastic changes, especially in glands [41]. This means that there were no dysplastic changes in the bronchial epithelium in both groups, but there was the possibility of changes in the gland and alveolar epithelium cells, which can result in the dysfunction and dysplasia of certain tissues.

One unexpected finding was the MUC-6 and NAPE-PLD expression in the hyaline cartilage. NAPE-PLD activity in cartilage has been detected in patients with osteoarthritis [53]. NAPE-PLD, in cooperation with Ca^2+^, induces anandamide (AEA) formation, whose level increases with disease severity [53]. This finding shows NAPE-PLD involvement in inflammation and the degradation processes of cartilage. According to other studies, the expression of MUC-6 is not detected in cartilage. Relevant studies have shown cartilage plasticity and complex adaptation mechanisms by expression of different unusual factors such as human beta defensin 2 (HBD-2), HBD-3 and cathelicidin (LL-37) [54,55], which suggest a possible lack of knowledge about cartilage’s potential involvement in inflammatory and adaptation processes.

An interesting finding was the increased secretion of IL-6 and IL-13 in all locations except connective tissue. The highest expression was detected in the cartilage and glands of both groups and in the alveolar epithelium and alveolar macrophages of the group with the stratified squamous epithelium.

IL-6 and IL-13 are produced by immune cells, fibroblasts and epithelial cells, and relevant studies have shown the ability of IL-6 to induce IL-13 synthesis and secretion [26,27,28,56,57]. Both cytokines have demonstrated a role in bronchial epithelium repair; they have also shown a significant role in lung disease development, such as COPD, asthma and involvement in lung fibrosis [27,57,58,59]. Our study demonstrated the increased secretion of IL-13 in the bronchial epithelium, with higher expression in the stratified squamosa epithelium group. Typically, IL-13 is associated with bronchial epithelial cell (especially glandular cell) hyperplasia, increased secretion of mucins, mucus metaplasia and eosinophil infiltration of the bronchial mucosa; however, the complete effects of IL-13 on the bronchial epithelium are unknown [60]. Our study demonstrates the possible involvement of IL-13 in changes of the respiratory epithelium to stratified squamous epithelium. Additional studies are necessary to identify possible IL-13 induced molecular mechanism involvement in bronchial epithelium changes to stratified squamous epithelium.

Moreover, we observed an increased secretion of IL-13 and IL-6 in the glands of both groups. Relevant studies have shown IL-13 involvement in the development of exocrinopathies and the remodeling of epithelial tissues [61]. Direct and indirect mechanisms are involved in the development of exocrinopathies by IL-13. Indirect mechanisms include prolonged stimulation of inflammation processes by immune cell activation, which leads to permanent triggering of the cells [62]. The direct influence of cytokines enhances the production of mucins and stimulates hypertrophy and hyperplasia of glandular cells [11,25]. The role of IL-6 is no less significant. IL-6 is involved in epithelial repair, and the permanent production of IL-6 leads to remodeling of epithelial tissue, not only in the bronchial epithelium, but also in glands [29,30,63]. This results in the hyperplasia of glandular cells and the increased production of mucins. IL-6 initially induces increased production of MUC-5; however, the persistent influence of IL-6 can provoke the expression of MUC-2 and MUC-6, which are associated with metaplasia and dysplasia in the lungs [10,29,30]. Both interleukins influence the tissue and induce adaptation mechanisms, which result in structural and functional changes like hyperplasia or metaplasia, and the increased secretion of mucins and other factors.

Although IL-13 and IL-6 are involved in a wide spectrum of processes in the lungs and produced by a number of different types of cells, it was interesting to find high expression of these cytokines in the hyaline cartilage of the respiratory system. Moreover, the expression of IL-6 and IL-13 was slightly higher in the pseudostratified epithelium group. The secretion of different factors by hyaline cartilage demonstrates its high adaptability to different situations; however, overexpression or prolonged expression of different factors can result in cartilage degradation. While vascular endothelial growing factor (VEGF) is involved in fetal hyaline cartilage development, VEGF has shown a degradational effect on cartilage [64]. Persistent inflammation results in decreased cartilage volume and increased degradation of cartilage proteoglycans in asthma patients, which led to stronger bronchospasms and more severe disease [65]. The expression of different antimicrobial peptides was also detected in hyaline cartilage [54]. These findings show the plasticity of cartilage and its possible involvement in the development of chronic diseases in the lungs.

As mentioned earlier, IL-6 and IL-13 are produced by immune cells and epithelial cells. In our study, we detected high expression of both interleukins in the alveolar epithelium and alveolar macrophages in the stratified squamous epithelium group. We detected an intercorrelation of IL-6 and IL-13 in alveolar macrophages and the alveolar epithelium and, moreover, a statistically significant difference was observed for IL-6 in alveolar macrophages in both study groups. IL-6 is involved in the repair processes of the epithelium, in chronic pulmonary disease development and induction of secretion of IL-13. Meanwhile, IL-13 mainly is associated with allergic asthma induction and has less involvement in chronic processes of the respiratory system [59]. However, relevant studies have shown increased synthesis and secretion of IL-13 by alveolar macrophages in lungs with fibrotic changes [66,67]. Synergistical cooperation and dominant production by immune cells of IL-6 and IL-13 leads to persistent expression of both interleukins. Furthermore, this prolonged expression of IL-6 and IL-13 by macrophages stimulates secretion of different factors in other cells (alveolar epithelium cells) and can result not only in fibrotic changes of the lungs but also in metaplastic or dysplastic changes of the epithelium.

The limitations of our study are associated with the relatively small subject groups. To obtain more accurate results, it is necessary to increase the number of subjects in each group, especially in the metaplastic epithelium group, and include more older patients. Moreover, it is necessary to include patients with a mucoidal epithelium and compare the expression of MUC-2, MUC-6, IL-6, IL-13 and NAPE-PLD in the mucoidal epithelium group with the pseudostratified and stratified squamous epithelium groups, which we plan to do in the future. Furthermore, correlations between age and gender should be promoted in future studies. In this study, only the immunohistochemistry method was used; it could be valuable to compare results by applying other methods such as ELISA, PCR and Western Blot in the future.

## 5. Conclusions

In the lungs with bronchial stratified squamous epithelium, regardless of age, alveolar macrophages were the main source of mucin (MUC-2, MUC-6), NAPE-PLD and interleukin (IL-6, IL-13) production. This demonstrates immune cells’ (especially alveolar macrophages) dominant involvement in the dysregulation of cell secretion and metaplastic changes. Meanwhile, the bronchial epithelium, which is the most sensitive to exogenous trigger factors, demonstrates no expression of MUC-2, MUC-6 and NAPE-PLD, which indicates the absence of dysplasia in the bronchial epithelium of both groups: the pseudostratified ciliated and stratified squamous epithelium groups.

Increased secretion of IL-13 in the stratified squamous epithelium group shows its involvement in metaplastic processes of the lungs; however, additional studies are needed to determine the IL-13 pathogenetic mechanism for metaplasia of the bronchial epithelium.

The detection of MUC-6, NAPE-PLD and interleukin (IL-6, IL-13) expression by bronchial hyaline cartilage demonstrates the significant adaptability and plasticity of this structure to the environmental changes via still unclarified pathogenetic mechanisms.

## Figures and Tables

**Table 1:** Relative number of MUC-2, MUC-6 and NAPE positive structures in the pseudostratified ciliated epithelium and squamous stratified epithelium.

No.	Sex	Age	MUC-2		MUC-6		NAPE-PLD			
			G	AM	C	AM	C	G	AE	AM
**Pseudostratified ciliated epithelium**										
**1**	M	16	0	0	0/+	0	0	0	0	0
**2**	M	19	0	0	0/+	0	0	0	0	0
**3**	M	19	0	0	0	0	0	0	0	0
**4**	M	29	+	0/+	0	0/+	++	0	++	+/++
**5**	F	55	0	0	0/+	0	0	0	0	0
**6**	M	56	0	+	0	+	+	0	+/++	++
**7**	M	61	0	0	0	0	0	0	0	0
**8**	M	64	0	0	0	0	0	0	0	0
**9**	F	85	0	0	0	0	0	0	0	0
**10**	M	94	0	+	0	+	0	0	0	0
**11**	M	94	0/+	0	0/+	0	0	+v	0	0
**12**	F	95	0	0	0	0	0	0	+/++	0
**Common mean value:**			0	0	0	0	0/+	0	0/+	0/+
**Stratified squamous epithelium**										
**13**	M	23	0	+	0	+	++	0	++	+/++
**14**	M	25	0	0	0	+	0	0	0	0
**15**	M	46	0/+	0	0	0	++	0	0	0
**16**	M	67	0	0	0/+	0	0	0	0	0
**17**	M	86	0	0	0	0	0	0	0	0
**18**	M	93	0	0	0	0	0	0	0	0
**Common mean value:**			0	0	0	0/+	+	0	0/+	0

Abbreviations: M, male; F, female; C, cartilage; G, glands; AE, alveolar epithelium; AM, alveolar macrophages; MUC-2, mucin 2; MUC-6, mucin 6; NAPE-PLD, N-acyl-phosphatidylethanolamine-hydrolyzing phospholipase D; 0—no positive structures in the visual field; 0/+—occasional positive structures in the visual field; +—few positive structures; +/++—few to moderate number of positive structures in the visual field; ++—moderate number of positive structures in the visual field.

**Table 2:** Relative number of IL-6 and IL-13 positive structures in the pseudostratified ciliated epithelium and squamous stratified epithelium.

Nr.	Sex	Age	IL-6						IL-13					
			BE	CT	C	G	AE	AM	BE	CT	C	G	AE	AM
**Pseudostratified ciliated epithelium**														
**1**	**M**	**16**	0/+	0	+	0	0	0	+	0	++	0/+	0	0
**2**	**M**	**19**	+	0/+	++/+++	++	0	0	++	+	++/+++	+	0	0
**3**	**M**	**19**	0/+v	0	+/++	0	+	0/+	+	0/+	++	+	++/+++	++/+++
**4**	**M**	**29**	0	0	0/+	0	+	0	0	0	++/+++	++	+/++	++
**5**	**F**	**55**	0/+	0	+	+/++	0	0	0/+	0	++/+++	+	0	0
**6**	**M**	**56**	+	0/+	0/+	0	+	++	+	0	++	0/+	++/+++	++/+++
**7**	**M**	**61**	0/+	0	0	0/+	0	0	++	0/+	0	++	0	0
**8**	**M**	**64**	0	0	++	0	0	0	+/++	0/+	+++	++	0	0
**9**	**F**	**85**	0	0	0	0	0	0	0	0	+	0	0	0
**10**	**M**	**94**	0/+	0	++/+++	0/+	0/+	0	+	+	+++	+/++	++/+++	+++
**11**	**M**	**94**	+	0	+/++	+/++	0	0	++	0	++/+++	+/++	0	0
**12**	**F**	**95**	0/+	0	0	0	++	++	0/+	0	0/+	+	++	++
**Common mean value:**			0/+	0	+	0/+	0/+	0/+	+	0/+	++	+	+	+
**Stratified squamous epithelium**														
**13**	**M**	**23**	0/+	0	0/+	+	++/+++	++	++	++	++/+++	0	++/+++	++/+++
**14**	**M**	**25**	0/+	0	0/+	0/+	++	++/+++	+/++	0/+	++	0/+	0	0
**15**	**M**	**46**	+	0	0/+	0/+	0/+	+	+	0	++	+	+	0/+
**16**	**M**	**67**	+	0	+/++	+/++	+/++	++	+/++	0/+	++	0/+	0	0
**17**	**M**	**86**	0	0	0	0	0	0	0/+	0	++	+	++/+++	++/+++
**18**	**M**	**93**	0/+	0	0/+	0	+/++	++	++/+++	0/+	++	0	++/+++	++
**Common mean value:**			0/+	0	0/+	0/+	+/++	+/++	+/++	0/+	++	0/+	+/++	+/++

Abbreviations: C, cartilage; CT, connective tissue, G, glands; BE, bronchial epithelium; AE, alveolar epithelium; AM, alveolar macrophages; IL-6, interleukin 6; IL-13, interleukin 13; 0—no positive structures in the visual field; 0/+—occasional positive structures in the visual field; +—few positive structures in the visual field; +/++—few to moderate number of positive structures in the visual field; ++—moderate number of positive structures in the visual field; ++/+++—moderate to numerous positive structures in the visual field; +++—numerous positive structures in the visual field.

**Table 3:** Mann-Whitney U test revealing statistically significant difference between pseudostratified ciliated epithelium and stratified squamous epithelium.

Detected Factors	Mann-Whitney U	Z-Score	*p*-Value
IL-6 in alveolar macrophages	12.5	−2.447	0.024

Abbreviations: IL-6, interleukin 6.

**Table 4:** Friedman test with pairwise comparison revealing statistically significant difference between different factors and different tissue locations in the pseudostratified ciliated epithelium group.

Marker 1	Marker 2	Z-Score	*p*-Value *
IL-13 in cartilage	MUC-2 in bronchial epithelium	−4.498	0.003
	MUC-2 in connective tissue	−4.498	0.003
	MUC-2 in cartilage	−4.498	0.003
	MUC-6 in alveolar epithelium	−4.498	0.003
	MUC-2 in alveolar epithelium	−4.498	0.003
	MUC-6 in glands	−4.498	0.003
	MUC-6 in bronchial epithelium	−4.498	0.003
	MUC-6 in connective tissue	−4.498	0.003
	NAPE-PLD in bronchial epithelium	−4.498	0.003
	NAPE-PLD in connective tissue	−4.498	0.003
	NAPE-PLD in glands	−4.185	0.012
	IL-6 in connective tissue	−4.023	0.025
	MUC-2 in glands	−3.930	0.037

Abbreviations: MUC-2, mucin 2; MUC-6, mucin 6; NAPE-PLD, N-acyl-phosphatidylethanolamine-hydrolyzing phospholipase D; IL-6, interleukin 6; IL-13, interleukin 13. *—with Bonferroni correction.

**Table 5:** Spearman’s rank correlation coefficient revealed correlations between the relative numbers of different factors in the pseudostratified ciliated epithelium group.

Strength of Correlation	Marker 1	Marker 2	Rho	*p*-Value
**Very Strong Positive Correlation**	IL-13 in alveolar epithelium	IL-13 in alveolar macrophages	0.991	<0.001
	NAPE-PLD in cartilage	NAPE-PLD in alveolar macrophages	0.983	<0.001
**Strong Positive Correlation**	IL-6 in alveolar epithelium	IL-13 in alveolar epithelium	0.894	<0.001
	IL-6 in alveolar epithelium	IL-13 in alveolar macrophages	0.863	<0.001
	IL-6 in cartilage	IL-13 in cartilage	0.825	0.001
	NAPE-PLD in cartilage	NAPE-PLD in alveolar epithelium	0.817	0.001
	NAPE-PLD in alveolar epithelium	NAPE-PLD in alveolar macrophages	0.796	0.002
	IL-6 in alveolar epithelium	IL-6 in alveolar macrophages	0.787	0.002
	NAPE-PLD in alveolar epithelium	IL-6 in alveolar epithelium	0.766	0.004
	MUC-2 in alveolar macrophages	IL-13 in alveolar macrophages	0.740	0.006
	MUC-6 in alveolar macrophages	IL-13 in alveolar macrophages	0.740	0.006
	MUC-2 in alveolar macrophages	NAPE-PLD in alveolar macrophages	0.732	0.007
	MUC-6 in alveolar macrophages	NAPE-PLD in alveolar macrophages	0.732	0.007
	MUC-2 in alveolar macrophages	NAPE-PLD in cartilage	0.711	0.009
	MUC-6 in alveolar macrophages	NAPE-PLD in cartilage	0.711	0.009
	IL-6 in alveolar macrophages	IL-13 in alveolar epithelium	0.704	0.011
**Moderate Positive Correlation**	MUC-2 in alveolar macrophages	IL-13 in alveolar epithelium	0.684	0.014
	MUC-6 in alveolar macrophages	IL-13 in alveolar epithelium	0.684	0.014
	IL-6 in cartilage	IL-13 in connective tissue	0.640	0.025
	IL-6 in bronchial epithelium	IL-6 in connective tissue	0.632	0.027
	MUC-6 in cartilage	IL-6 in glands	0.631	0.028
	IL-6 in alveolar macrophages	IL-13 in alveolar macrophages	0.608	0.036
	MUC-2 in glands	NAPE-PLD in glands	0.604	0.037
	IL-6 in bronchial epithelium	IL-13 in bronchial epithelium	0.580	0.048

Abbreviations: MUC-2, mucin 2; MUC-6, mucin 6; NAPE-PLD, N-acyl-phosphatidylethanolamine-hydrolyzing phospholipase D; IL-6, interleukin 6; IL-13, interleukin 13.

**Table 6:** Spearman’s rank correlation coefficient revealed correlations between the relative numbers of different factors in the stratified squamous epithelium group.

Strength of Correlation	Marker 1	Marker 2	Rho	*p-*Value
**Very Strong Positive Correlation**	IL-13 in alveolar epithelium	IL-13 in alveolar macrophages	0.953	0.003
	IL-6 in alveolar epithelium	IL-13 in connective tissue	0.939	0.005
**Strong Positive Correlation**	MUC-6 in alveolar macrophages	IL-6 in alveolar epithelium	0.840	0.036
	IL-6 in alveolar epithelium	IL-6 in alveolar macrophages	0.832	0.040
	IL-6 in bronchial epithelium	IL-6 in cartilage	0.822	0.045
	IL-13 in bronchial epithelium	IL-13 in connective tissue	0.814	0.049

Abbreviations: MUC-6, mucin 6; IL-6, interleukin 6; IL-13, interleukin 13.

## Data Availability

Not applicable.
